# Biodiversity Change in Cultural Landscapes—The Rural Hotspot Hypothesis

**DOI:** 10.1002/ece3.70811

**Published:** 2025-01-08

**Authors:** Carsten Neumann, Robert Behling, Gabriele Weiss

**Affiliations:** ^1^ Helmholtz Centre Potsdam GFZ German Research Centre for Geosciences Potsdam Germany; ^2^ Ecostrat GmbH Berlin Germany

**Keywords:** agricultural intensification, backyard habitats, biodiversity loss, cultural landscape, satellite imagery, species decline, time‐series analyses

## Abstract

A dramatic decrease of biodiversity is currently questioning human‐environment interactions that have shaped ecosystems over thousands of years. In old cultural landscapes of Central and East European (CEE) countries, a vast species decline has been reported for various taxa although intensive land cultivation has been reduced in favor of agroecological transformation, nature conservation and sustainable land management in the past 30 years. Thus, in the recent history, agricultural intensification cannot solely be discussed as the major driver controlling biodiversity. In cultural landscapes, we state that drivers and pressures mainly emerge from the backyards of rural settlements that act as interconnected rural hotspots and therefore form an ecological metapopulation in which small‐scale backyard habitats are capable of preserving and exchanging species pools of the historical cultural landscape. We further argue that shifting sociocultural norms significantly affecting the survival of source populations in rural hotspots and drastically limit their dispersal pathways, which triggers the degradation of the rural metapopulation in recent times. Pressures of cultivation shift, landscape decoupling, structural homogenization, and use of technology and agrochemicals are identified as backyard ecological drivers negatively affecting biodiversity preservation, particularly in the surrounding rural landscape. Spatiotemporal dimensions of backyard pressures involving material fluxes, species exchange and retention, alternation of site conditions, and local genetic adaptation are delineated for different backyard features, including building structures, gardens, lawns, and paved grounds. Finally, we propose a future research agenda to quantify effects and trends of rural hotspots and followed patterns of altered species dynamics. We give an example on the use of satellite time series to remotely map rural backyard habitats and reveal significant spatiotemporal trends induced by small‐scale human behavior that may lead to a new socioecological perception and stimulate actions to shape ecological dynamics emerging from the backyards of human settlements.

## Graphical abstract

In the recent history of cultural landscapes, agricultural intensification can no longer be discussed as the major driver controlling biodiversity. There is evidence that rural settlements act as interconnected rural hotspots and therefore form an ecological metapopulation in which small‐scale backyard habitats are capable of preserving and exchanging historically estabilshed species pools. New technologies, e.g., remote sensing time series analyis, will help to monitore the ongoing degradation of the rural metapopulation to stimulate future actions against biodiversity loss.

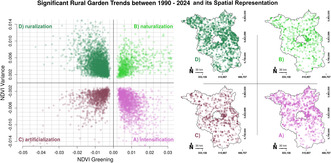

## 1 Introduction—The Cultural Context of Biodiversity Change

The world is observing a strong decline in biodiversity, which follows various anthropogenic ecosystem interferences. Recent knowledge about biodiversity loss is often referred to the decline of species numbers or species biomass owing to habitat loss, as indicated by time‐series records of faunistic and floristic surveys, which show strong negative trends worldwide (Almond, Grooten, and Peterson 2020; Lister and Garcia 2018; Pereira et al. 2024; Sánchez‐Bayo and Wyckhuys 2019; Wagner et al. 2021). Many factors are described on various taxa, i.e., pollution, hunting, land take, and habitat fragmentation through urbanization, colonization, industrialization, and/or agricultural intensification where new human settlements carve large niches into natural landscapes and convert natural ecosystems into anthropogenic ones (Abudulai et al. 2022; Ritter 2011; Vitousek 1997). However, biodiversity decline is also present in (post‐)industrial societies of, e.g., Europe where human land‐use practices have already initiated ecosystem change at multiple scales over thousands of years and have finally shaped quasi‐non‐natural, widespread cultural landscapes (Agnoletti 2014; Antrop 2004). Although many ecosystems in Europe are thus affected broadly by agriculture and forest clearance since the Neolithic period, where today no untouched wilderness exists, rates of biodiversity loss are generally comparable to worldwide trends. Even for a hundred years now, nature conservation by designing ecological networks has become an important part of European land‐use practice defining protected areas in which natural habitats become restored (Bingham et al. 2021; Jongman 1995). The historical background of cultural landscapes thus may lead to complex interactions in a human‐environmental system that still make it difficult to disentangle the underlying mechanisms of biodiversity change and explain recent patterns of biodiversity decline.

In the European cultural landscape, trends and future dynamics of biodiversity are connected to an inherent long‐term human presence forcing a historic coevolution of species depending on human demography and technological development with different responses on genetic, taxonomic, and functional levels of biodiversity. Consequently, biodiversity loss cannot solely be linked to processes of nature‐to‐culture conversion. In fact, agricultural transformation once led to faunal and botanical diversification through anthropogenic land‐use change from 6.000 BP onward when agriculture was imported to Europe. It was the emergence of the European rural landscape in which agriculture initiated steady habitat transformation between shifting land cultivation and livestock keeping in close proximity to human settlements that were founded in their current arrangement during medieval times. Closely interconnected habitats with active nutrient relocation between extensive small‐scale land use regimes of farmlands, (wood‐)pastures, or heathlands accompanied by large forest clearance (between 80% and 90% of mixed‐temperate forests) finally lead to a significant biodiversity peak at the beginning of the 19th century (Bignal and McCracken 2000; Fukarek 1979; Sukopp and Trepl 1987). Hence, statements such as “agriculture is the largest contributor to biodiversity loss” or “agriculture destroys biodiversity” (Dudley and Alexander 2017) are not generally true, particularly not in traditional cultural landscapes where shifting biodiversity baselines are an inherent characteristic of close human‐environment interactions (Agnoletti 2014; Cevasco and Moreno 2012). Currently, biodiversity loss is hence regarded as a species decline from the last early 19th‐century peak as a consequence of modern agriculture that facilitated the process of agricultural intensification at different stages nonuniformly distributed in Europe. Intensification comprises the substitution of traditional grazing cycles by stall‐feeding with newly introduced leguminous feed crops (e.g., *Trifolium pratense* , *Medicago sativa* ), the implementation of mineral fertilizer that progressively replaced organic fertilizer such as dung, compost or sod cuts and the utilization of technology in form of agricultural machinery for land cultivation (Chorley 1981; Thompson 1968; Van Zanden 1991). Productivity growth was further accelerated following the unification of fields and site factors through technological management that reduced biodiversity due to structural homogenization, decreasing ecotone areas or reduced disturbance dynamics (Clough, Kirchweger, and Kantelhardt 2020; O'Brien, Prados, and La Escosura 1992; Van Zanden 1991). Agricultural intensification was rapid particularly during socialist land collectivization in Central‐East Europe (CEE) (excluding Poland) between 1945 and 1965, initiating a wide application of pesticides, the development of advanced plant breeding, and an increased application of mineral fertilizer, which further increased farmland productivity in the second half of the 20th century (Skokanová, Falt'an, and Havlíček 2016).

### 1.1 A Contradicting View on Biodiversity Decline and Agricultural Intensification

The key factors triggering biodiversity loss from the beginning 19th century onward can be clearly defined as agricultural intensification mainly changing landscape architecture, decoupling nutrient cycles, introducing technological land management and distributing agrochemicals such as fertilizers and pesticides (Emmerson et al. 2016; Geiger et al. 2010). Interestingly, from a recent perspective, it should be recognized that negative effects of human‐environment interactions have either finalized for decades or have significantly reduced since the early 1990s of the 20th century in CEE countries: Chemical fertilizer consumption was drastically reduced by 50%–74% from 1989 to 2019 (BMEL 2021; Stoate et al. 2009; Sutcliffe et al. 2015), while pesticide usage has exhibited a slightly negative trend over the last 30 years (BMEL 2021; EU 2021). Field sizes were decreasing due to the splitting of large parcels and land abandonment in the process of postsocialist privatization and decollectivization (Griffiths et al. 2013; Prokopová et al. 2018; Sabates‐Wheeler 2002; Václavík and Rogan 2009). In a systematic review (Plieninger et al. 2016) revealed that between 1990 and 2015 the most prominent driver of European landscape change was land abandonment/extensification. Moreover, biodiversity‐promoting measures (e.g., flower strips, stepping stone habitats, agroforestry), ecological‐organic farming, precision agriculture, and smart farming have been strongly expanded and were steadily replacing conventional agricultural practices toward a new paradigm to foster sustainable agroecosystems (Kernecker et al. 2020; Moudrỳ et al. 2018; Pawlewicz et al. 2020; Tscharntke et al. 2021). At the same time, atmospheric pollution negatively affected plant species diversity by deposition of, e.g., nitrogen and sulfur oxides were considerably reduced (Drosihn 2021).

From the early 1990s an increasing agroecological transformation, reduced inputs of harmful substances and more deintensified land use practices can be concluded for the European cultural landscape that is, however, crucially contrasted by a dramatic and ongoing decrease of different biodiversity indicators in the last 30 years. For example, the prominent Krefeld study reports on a strong decline in total flying insect biomass by 76% between 1989 and 2016 in western Germany (Hallmann et al. 2017). The results are confirmed across various gradients of land‐use intensities in, e.g., Germany where biomass decline was reported for arthropods by 67% (2008–2017) (Seibold et al. 2019), for hoverflies by 89% (1989–2014) (Hallmann et al. 2021), for nocturnal moths by 48% (1989–2018) (Roth et al. 2021), or for carabid counts by 60% (1989–2011) (Skarbek, Kobel‐Lamparski, and Dormann 2021). Species richness of butterflies is reduced by 20% (1990–2013) (Habel et al. 2016) or by 10% (2005–2015) (Rada et al. 2019), of carabids by 31% (1989–2011) (Skarbek, Kobel‐Lamparski, and Dormann 2021) or by 25% (1994–2017) (Homburg et al. 2019). Further drastic decline can be observed in counts of farmland birds by 40% (1990–2018) (Kamp et al. 2021), whereas species richness of vascular plants is only slightly decreasing by 6% (1990–2008) (Wesche et al. 2012) or by 1.9% per decade (1988–2017) (Eichenberg et al. 2021). All long‐term records are summarized for Germany, although there is strong evidence for an ongoing species decline in all European countries (Burns et al. 2021; De Heer, Kapos, and Ten Brink 2005; Rigal et al. 2023).

Although agricultural intensification is the most discussed driver behind recent decline of insects, avifauna, and plant taxonomic and functional diversity (Carmona et al. 2020; Habel, Samways, and Schmitt 2019; Rigal et al. 2023; Rumohr et al. 2023), the temporal discrepancy between recorded negative biodiversity trends and actual statistics about indicators that determine agricultural intensification such as usage of fertilizer, pesticides, mechanical tillage, land unification, etc., becomes obvious in cultural landscapes, such as Europe. In fact, cereal yields as prominent intensification indictor are stagnating for all CEE countries since 1990 (Brisson et al. 2010; Liira et al. 2008). In this respect, the fundamental incongruity is that most extinctions of species should already have occurred due to realized habitat loss long time ago, which is usually true for short‐lived specialist species, e.g., insects, annual plants and for small fragmented habitats (Hanski and Ovaskainen 2002; Krauss et al. 2010). Conversely, ecological legacy effects might still be pending, most likely in cases of long‐lived species, e.g., perennial plants, mammals on large and connected habitats where persistent extinction is often described as (co‐)extinction debt in a metapopulation context (e.g., Culbert et al. 2017; Deák et al. 2021; Kuussaari et al. 2009; Löffler, Poniatowski, and Fartmann 2020). In fragmented landscapes, delayed (co‐)extinction is facilitated by increasing habitat numbers and area, decreasing habitat isolation and temporally close habitat destruction time (with variable time spans of debt duration between 5 and 1000 years) (Figueiredo et al. 2019; Hanski and Ovaskainen 2002; Hylander and Ehrlén 2013). It is clearly evident that biodiversity dynamics can only be understood against this background of patterns and processes concerning the dynamic configuration of remaining habitat fragments in a landscape context. In order to establish a causal link between ecosystem change and biodiversity loss it is thus of utmost importance to understand agriculture as part of a spatiotemporally interconnected cultural landscape that particularly includes rural settlements as old remnants of a historical highly diverse background, their ecological integrity and multitrophic, multispecies exchange pathways in which historic and recent anthropogenic activities have evolved over thousands of years and thus stimulating biodiversity over complex agroecological networks. For this purpose, we frame the hypothesis of a degrading rural metapopulation where villages act as remaining rural hotspots that are currently under pressure considering recent sociocultural change and outline a research agenda to better monitor the influence of cultural drivers and gain knowledge to address conservation measures for future biodiversity control.

## 2 The Rural Hotspot Hypothesis

In a rural landscape context, the survival of local species pools can only be guaranteed over long time periods by connected networks of source habitats that are embedded in a metapopulation structure and thus permanently provide recolonization pathways. We state that backyard habitats in rural settlements act as anchor points for a historically interconnected metapopulation that represents the ecological network of a grown cultural landscape. Therein, backyard habitats consist of green features such as home gardens, lawns, trees, and shrubs and also include structural features such as buildings, playgrounds, walls, animal enclosures, ponds, tracks, debris, and storage areas. Their small‐scale feature variation provides diverse ecological niches for plants and animals that are capable of generating unique and stable habitats even within an intensively used agricultural landscape. For example, some wild birds are shown to occupy mainly small and stable breeding territories in south‐east England's gardens (71.5%) even though these habitats only cover 2% of the total area (Mason 1998). In intensively used areas, rural settlements already have been recognized as the most important habitat for the conservation of many sedentary farmland birds (Cannon 1999; Havlíček et al. 2021; Rosin et al. 2016; Šálek, Bažant, and Żmihorski 2018). Moreover, home gardens exhibit a remarkably high small‐scale plant species diversity in comparison to surrounding seminatural habitats (Thompson et al. 2003). Backyard habitats are characterized by generally low population sizes and related microhabitats owing to the irregular practice of maintenance and abandonment. This way, private gardens supply a wide range of diverse resources, particularly for pollinating insects that accumulate in hotspots of high abundances and species numbers against a background dominated by intense farmland (Samnegård, Persson, and Smith 2011). There is evidence that also mammals benefit from the introduction of supplementary food and structure resources leading to higher richness, diversity, and abundance compared to wild sites (Hansen et al. 2020; Parsons et al. 2018).

**FIGURE 1:**
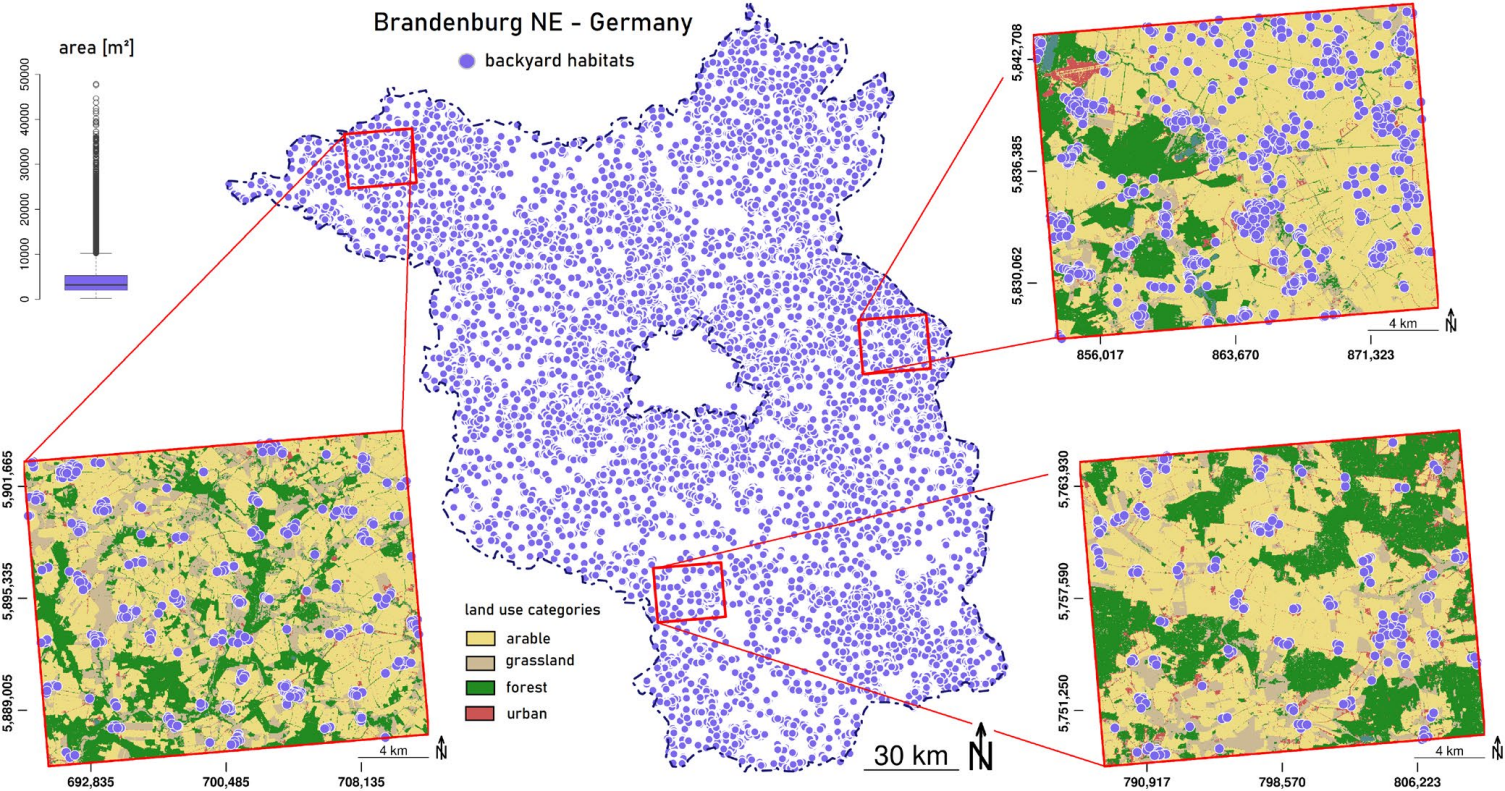
Spatial distribution of backyard habitats, shown as polygon centroids of garden areas, embedded in a rural landscape that is mainly agriculturally used; boxplot of polygon areas derived from biotope mapping in 2009, Landesamt für Umwelt (LfU) und Landesvermessung und Geobasisinformation (LGB) Brandenburg, Germany.

Although the total area amount of backyard habitats is generally small, their distribution is ubiquitous and relatively dense, enabling small linkage paths, across all types of land use (Figure 1). This way, backyard habitats are able to form ecological networks for holding sink and source dynamics of species dispersal in a metapopulation framework (Rudd, Vala, and Schaefer 2002). They represent nodes of high structural complexity and multifunctional diversity in which genetic diversity of the historical cultural landscape has been preserved over centuries (Galluzzi, Eyzaguirre, and Negri 2010). In this regard, they are considered as hotspots for agro‐biodiversity since they are predominantly remnants of historic rural settlements and related extensive land use practice. As an example of historical continuity, scattered old trees in traditional meadow orchards nowadays representing keystone ecological structures for the preservation of biodiversity that particularly affect surrounding areas in agricultural landscapes (Horak et al. 2013; Manning, Fischer, and Lindenmayer 2006; Plieninger et al. 2015). Rural settlements thus generate highly structured and interconnected backyard habitats that have already proven to control multiple levels of biodiversity, considerably affecting the landscape in which they are integrated.

Significant sociocultural changes have impacted the rural metapopulation with dramatic consequences for farmland biodiversity that have particularly begun for CEE countries in the early 1990s after the change of communist political system and followed EU accessions. After 1990, modernization of farmstead and villages have shown to entail significantly higher contributions to bird species decline than agricultural intensification itself (Rosin et al. 2021), although there is still ongoing debate about underlying mechanisms to also link field nesters decline (Hertzog et al. 2022). In the past three decades, an ongoing harmonization of socioeconomic conditions between urban and rural areas have most likely further affected human‐environment interactions in backyard habitats that are still relatively unexplored. In rural settlements, increased welfare induces improved living standards which in turn change social norms that are controlling human‐induced environmental pressures such as effects of increased prosperity on building constructions, renovations and farmstead design as well as changing patterns of work and organization of time on animal keeping, cultivation practice and garden use. Their implications for structural and functional modifications of backyard habitats and arising consequences for biodiversity on the landscape scale are still being underestimated. Thus, revealing the spatiotemporal dimensions of drivers and pressure indicators in the context of backyard metapopulations can be the basis for a new research agenda to support and schedule future conservation measures that involve social, economic and environmental factors into an integrated rural landscape approach.

## 3 Drivers and Pressure Indicators for Degrading Rural Hotspots

### 3.1 Use of Agrochemicals

An uncontrolled availability and hence increased household applications of mineral fertilizers gradually decreases fine‐scale backyard habitat diversity by aligning nutrient conditions, particularly converting nutrient‐poor niches to highly productive sites in private gardens. Artificial fertilizers undermine historically established and closely linked nutrient cycles that were previously activated from litter or dung composting. Currently, many gardens are already considered as over‐fertilized (Cameira, Tedesco, and Leitao 2014). In particular, lawns are exposed to massive amounts of fertilizer. It was shown that the per hectare input of fertilizers to maintain vital and robust lawns can exceed chemicals added for food production in the U.S. (Robbins and Sharp 2003). There is evidence that nitrogen and phosphorus leaching from urban lawns affects eutrophication of groundwater and open water bodies (Groffman et al. 2004; Sharma et al. 1996). Comparable studies are surprisingly rare for CEE rural landscapes. (Kalmykova et al. 2012) remarks that there are no statistics on mineral fertilizer use for nonagricultural purposes to determine urban phosphorus budgets in Sweden. There are a few studies pointing to the still underestimated role of household fertilizer applications, e.g., fertilizing in Austrian gardens contributes to 12% of environmental nitrogen flows (Pierer, Schröck, and Winiwarter 2015) or vegetable gardening with an area coverage of 3.5% is responsible for 27% of total nitrogen eluviation (Kliebsch, Müller, and Van Der Ploeg 1998). However, up to date, there are no statistics available about temporal trends of private fertilizer use and its implications for species loss on a landscape scale.

Furthermore, systematic empirical surveys on trends and effects of private pesticide usage are also missing, particularly with regard to rural biodiversity (Robbins, Polderman, and Birkenholtz 2001). It is known that pesticide usage on private lawns or gardens has strong negative impacts on plant species richness (Bertoncini et al. 2012), soil microfauna (Byrne and Bruns 2004), and flower visiting insects (Muratet and Fontaine 2015), with even potential threats to human food safety and health (Meftaul et al. 2020). However, their use is only partly regulated (e.g., prohibition of Glyphosate for private use, September 2021, Germany) and can hardly be monitored by environmental agencies. An increased awareness of negative effects of agrochemical applications can only be achieved by shifting social norms toward an increasing motivation for a moral responsibility and reconnectivity to nature that confesses backyards as wildlife refuges (Goddard, Dougill, and Benton 2013).

### 3.2 Cultivation Shift

There are potential socioeconomic drivers to facilitate land use change from private cultivation (food gardens, flower beds, orchards) and animal keeping (chicken yards, hay barns, and stables, feeding grounds) toward extended monofunctional areas (lawns, paved grounds, gravel yards). Such areas can be less labor‐intensive since they fulfill mainly esthetic functions and allow for uniform management. In this regard, unmanaged ruderal vegetation and small deposit niches such as woodpiles, dung, and compost heaps or debris areas are consequently removed to homogenize new esthetic standards and replace forms of land use that are no longer essential for life care. The substitution of cultivation by aestheticization represents an ongoing leveling of rural–urban gradients, particularly with regard to forms of labor, household incomes, industrial food production, and private lifestyle concepts (holiday travels, digital media entertainment, and social representation). In general, the time held back for backyard management and active use is continuously reduced as a direct consequence of the socioeconomic alignment of living standards and lifestyle requirements between rural and urban settlements. Although, there is a known trend of increased suburbanization or periurbanization since the early 1990s in CEE countries (Shaw, van Vliet, and Verburg 2020; Szmytkie 2020), the effective range of changing lifestyle extension into remote rural villages is still unclear. Their negative effects on ecological networks and species pools, however, are highlighted (Holgerson et al. 2018; Jokimäki, Suhonen, and Kaisanlahti‐Jokimäki 2021; Rosin et al. 2021). One of the most vivid symbols of shifted social norms reflecting modern lifestyle is the lawn (Ignatieva et al. 2015). Its extent is growing, e.g., lawns have almost doubled from 12.6% to 22.5% relative cover between 1960 and 2015 in three Swedish cities (Hedblom et al. 2017). However, spatiotemporal trends of rural lawn areas and their managed plant communities are rarely documented although there is evidence that floristic lawn diversity is influenced by socioeconomic drivers (Wheeler et al. 2017). Recently, further trends have arisen to replace living lawns with synthetic grass for enlarging clean and easy‐to‐maintain green areas, particularly in urban landscapes (Francis 2018).

One important aspect of aestheticization is the introduction of non‐native plants into backyards. The magnitude of global exchange and hence local availability of exotic species, particularly ornamental plants for private garden arrangements, is rapidly increasing following higher risks of negative ecological impacts such as biotic homogenization (Blouin, Pellerin, and Poulin 2019; Simberloff et al. 2013; Van Kleunen et al. 2015). Since plants in their native environment provide highly diversified habitats for many insects, birds, and mammals due to long‐term coevolution, their replacement by non‐native plants may entail species loss if pollinator behavior is not adaptable or opportunistic (possibility of home range expansion) (Burghardt et al. 2010; Pardee and Philpott 2014; Tallamy, Narango, and Mitchell 2021) or even leads to population collapse if non‐native plants become dominant (> 30% plant biomass) (Narango, Tallamy, and Marra 2018). Recently, a new ecological paradigm has been discussed recognizing non‐native plants as additional pollinator resources, particularly for generalist species in temperate zones, when native plants become scarce at the end of the growing season (Koyama et al. 2018; Salisbury et al. 2015; Staab, Pereira‐Peixoto, and Klein 2020). More evidence is needed to understand the evolutionary history of food webs that has been established for backyard plant species compositions, particularly with regard to coevolving specialist species.

Paved grounds are an extreme example of intended cultivation shift and induced habitat loss through soil sealing in backyards. There is evidence of increased soil sealing rates also in rural areas by 20%−25% (1994–2006) in Italy (Munafò, Salvati, and Zitti 2013), by 33% (1987–2013) in Flanders, Belgium (Vanderhaegen and Canters 2016) or an average loss of land of 0.6% (2006–2012) in periurban areas across Europe (Naumann et al. 2019). However, in the underlying studies, there is no distinction made between patterns of urbanization leading to soil sealing through land taken by buildings or infrastructure and backyard processes stimulated by internal cultivation shifts. There seems to be a general confusion in defining and monitoring the term soil sealing, which is often interchanged with urban growth, neglecting fine‐scale land conversion processes in the backyards of settled areas. Such processes, addressed as “hidden urbanization” can result in a strong increase in impervious surface (by up to 56% between 1997 and 2016) as shown in Lower Saxony, Germany (Strohbach et al. 2019). Until now, there are no systematic studies available about temporal trends of backyard cultivation shifts potentially accelerating soil sealing rates in rural landscapes of CEE countries.

### 3.3 Landscape Decoupling

**FIGURE 2:**
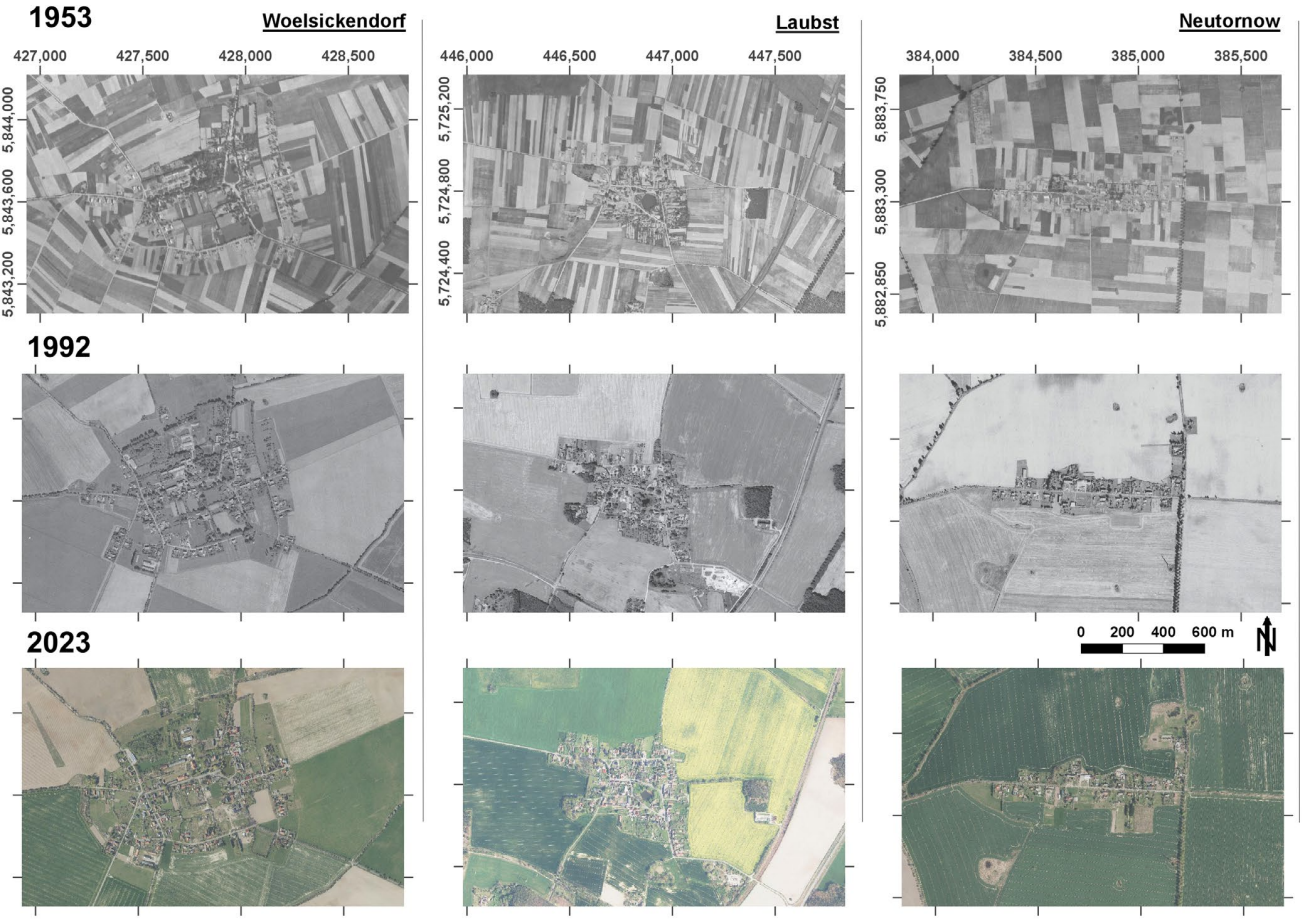
Changing village landscape integration on the basis of digital orthophotos comparing the years 1953, 1992, and 2023 in Brandenburg, Germany. Former continuous integration of village backyard cultivation and subsistence farmland was already fully replaced by segregated boundaries between rural settlement areas and the surrounding large, uniform agricultural fields in 1992. While field unification remains constant, rural settlement areas change in extent, building density, and form of use from 1992 to 2023.

To enable source populations that can preserve and spread species in a metapopulation context, backyard habitats need to be integrated into a metapopulation's dispersal network. Spatial connectivity and possible dispersal pathways between subpopulations play an important role in the stability and persistence of metapopulation systems. A continuous integration of backyards into the surrounding landscape matrix, hence, enhances network continuity by establishing interlinked keystone habitats and triggering species exchange. However, due to different isolation effects, backyards become increasingly decoupled from their surroundings, in particular as a recent phenomenon in rural areas (Figure 2). Backyard isolation can be physical through property boundaries of hedges, fences, or walls to intentionally protect and mark the ownership of land. They act as less permeable barriers against the movement of reptiles, amphibians, or mammals. For example, the frequency of Common Toad, Slow‐worm, and Grass Snakes occurrence is negatively correlated to artificial boundary features based on 3806 surveys in British gardens (Humphreys et al. 2011). In Australian suburban areas, backyard faunal wildlife activity exhibits the strongest negative effects when the fence material is closed (Fardell, Pavey, and Dickman 2022).

There is, furthermore, a functional decoupling when nutrient cycles are broken (e.g., litter input from the rural environment for animal bedding and garden fertilizing), dispersal paths are interrupted (e.g., free animal movement between property boundaries and villages) or subsistence farming is abandoned (e.g., individual land management for food production). The separation of functions between backyards and the surrounding landscape is directly limiting dispersal options by decreasing habitat continuity beyond the boundaries of rural properties. It was shown that plant species diversity on grasslands is significantly associated with the distance to the nearest historical village (unimodal relationship to 19th‐century locations), but not to present‐day villages, indicating a strong influence of traditional grazing practice to habitat connectivity reflecting the movement patterns of livestock in the historical landscape (Reitalu et al. 2010). In contrast, ornamental lawns are species‐poor, mostly comprising the same species composition, and thus do not represent native environments in which they are planted (Ignatieva et al. 2015). Arthropod diversity, abundance, and community composition are consequently higher with fewer lawn or grass‐free lawns, but also with larger vegetable gardens and more cover by woody plants that represent their natural background (Pardee and Philpott 2014; Smith et al. 2015).

Structural isolation additionally occurs in cases where a functional separation is supplemented by diverging management in which vegetation growth and phenology are detached from abiotic, natural site factors by implementing artificial irrigation, fertilization, illumination, and pest control. Decoupled environments of novel site compositions (e.g., altered seasonality and day/night periodicity) do not necessarily provide natural source populations but have the potential of creating isolated subpopulations through genetic local adaptation (Homola et al. 2019; Merckx et al. 2021). In fact, genetic shifts between species beyond the range of phenotypic plasticity can create genotypes that are incompatible with their original site conditions. Genetic change, thus, may not disperse into the natural environment back again to avoid developmental traps if, e.g., photoperiodic thresholds for controlling life‐cycle development or temperature thresholds regulating growth mismatch actual background cues.

### 3.4 Structural Homogenization

In the past decades, lifestyle factors, sociocultural norms, and the socioeconomic background have changed in traditional rural environments of CEE countries. Normative standards of cleanliness and representativeness are developing in step with growing prosperity. Processes of urbanization additionally spreading into the countryside while remote villages are affected by population loss and aging (Antrop 2000). As a consequence, backyards have become functionally homogenized due to village modernization, renovation, and reorganization of human land use interactions affecting farmland biodiversity in rural landscapes (Antrop 2000; Rosin et al. 2021; Żmihorski et al. 2020). This contrasts the fact that old farmsteads with animal and plant production are key habitats and provide biodiversity hotspots for farmland birds (Hiron et al. 2013; Rosin et al. 2016; Šálek, Bažant, and Żmihorski 2018). Also, floral diversity and related arthropod species richness can be bound to small‐scale variable microhabitat conditions of human‐made artifacts as described in wall ecology (Francis 2011) or animal—building interactions (de Wilde and de Souza 2022; Meier, Raps, and Leistner 2020). Moreover, the multitude of backyard features such as wood stacks, compost and debris heaps, leaf piles, ponds, trees, hedges, gardens, or areas for animal husbandry create an integrated small ecosystem with specialized nutrient, edaphic, microclimatic, and biotic conditions that provide a wide range of resources for biodiversity regulation (Davies et al. 2009; Galluzzi, Eyzaguirre, and Negri 2010). A structural homogenization can lead to local species extinction. However, the structural variety and spatial heterogeneity of backyard features as microhabitats for plants and arthropod species and for bird nesting are still being neglected by conservation policies.

Today, the removal of nesting sites in modern and renovated building architecture and the decrease of foraging grounds due to structural homogenization are identified as the main causes of an ongoing bird species decline (50% decline in building nesters, e.g., sparrows, swallows, owls in new and renovated houses) (Rosin et al. 2020). The composition of traditional village flora is still undergoing a considerable trend of homogenization toward common ubiquists that are expected in urbanized areas, comparing the periods 1980–1983 and 2004–2005 in North Rhine Westphalia, Germany (Huwer and Wittig 2013) or detecting a species decline by 31% between 1976 and 2012 in Hessen, Germany (Gregor et al. 2016). Even though the floral diversity of backyards may be still high, there is evidence that variation in structural metrics, for example, tree height and density, can be lower between yards than among natural areas, as shown in North American yards (Pearse et al. 2018). There is still a great need for research particularly, revealing temporal trends of backyard structural transformation along with social drivers that influence management decisions and alter ecological outcomes and ecosystem services in an integrative socioecological system (Cook, Hall, and Larson 2012; Hostetler 2021).

### 3.5 Use of Technology

Technology and automation have already entered backyards introducing new practices of intensive management. A recent development are robotic and autonomous mowing machines with expected annual growth rates of 21.9% (Grand View Research 2019). In order to enable a forward movement, such systems need to keep vegetation below an optimal cutting height resulting in almost permanent mowing (1–3 times per week) (Hossain et al. 2022; Sportelli et al. 2021). Robotic lawnmowing obviously entails drastic consequences for species diversity since the number of spontaneous flowers and adapted pollinators crucially depend on the mowing frequency. Mowing less frequent provides more time for flower development which results in a significant diversification of plant community composition (Chollet et al. 2018; Sehrt et al. 2020) and, hence, increases, e.g., bee species richness (Lerman et al. 2018; Wastian, Unterweger, and Betz 2016) or arthropod biomass and species numbers (Wintergerst et al. 2021). It was shown that heteropteran biodiversity was reduced by 50% in each monthly mowing event on urban grasslands (Unterweger, Rieger, and Betz 2017). Furthermore, a decrease of traditional heterogenous mowing operations (spatiotemporal asynchrony) negatively affects survival rates and resource availability for rural hay meadow invertebrates (Cizek et al. 2012).

Automatic irrigation systems have the potential to homogenously distribute water resources for maintaining high productive green backyard areas. Regular water provision, thus, contrast less productive xeric sites such as dry grasslands that are known to bear high species numbers due to higher competitive pressures (Cook and Faeth 2006). Irrigation can further increase grass density and evaporation rates, which leads to temperature modifications by shading and cooling. Such microclimate alterations can affect the development of thermophilous insects (Andrey, Humbert, and Arlettaz 2016; Humbert, Delley, and Arlettaz 2021); however, the mechanism impacting arthropod communities are still poorly understood, particularly with regard to rural backyards. Many ecological implications behind the advancement of modern management practice into backyards are still understudied. This regards for example noise pollution and gas emission from application of machinery or potentially negative effects of plastics for, e.g., bed borders or weed foils.

## 4 Research Agenda

### 4.1 Quantitative Analyses of Rural Hotspot Dynamics

According to the large number of various drivers that have been put pressure on different levels of biodiversity in backyard habitats, rural hotspots may have a significant impact on biodiversity dynamics in complex cultural landscapes. Empirical evidence is needed to determine the actual magnitude of their contribution to biodiversity change within the last three decades, particularly in comparison to agricultural land use, and hence to increase our potential influence to promote biodiversity from the backyard of rural settlements. Against this background, in order to identify and describe the rural hotspots and to delineate the rural metapopulation under changing socioeconomic backgrounds and arising management practice, local‐scale, spatiotemporally explicit, and temporally repeated observations of rural backyard habitats, their regional‐scale connectivity and their cultural‐historical genesis as part of a rural landscape are urgently required. For this purpose, direct measurements and field surveys of geochemical fluxes (e.g., the amount and type of fertilizer and pesticides used), of intensities of physical change (e.g., niche deconstruction from buildings, renovations, pavements), of recent and retained species diversity (e.g., species composition of lawns, trees, shrubs, gardens, soil seed bank reservoirs, and faunal surveys of, e.g., birds, insects, mammals) and of plant traits (vitality, phenology, life‐cycle, numbers of flowers and fruits, growth) have to be combined with recent and historic social statistics of socioeconomic factors (e.g., household incomes, employment) and sociocultural factors (e.g., environmental perception, time management, education) but also need to consider habitat continuity in a metapopulation network in which species dispersal beyond backyard borders are mapped analyzing habitat connectivity and material fluxes that extent backyard processes into the surrounding landscape. Since backyard habitats are part of legally protected spaces of privacy, citizen science projects have the potential to advance scientific surveys that can be complemented by environmental data collected by amateur naturalists. Ecological applications for citizen scientist are already versatile (Silvertown 2009), e.g., map trace metal contaminants in garden soils (Taylor et al. 2021), report problematic invasive plants (Dehnen‐Schmutz and Conroy 2018; Encarnação, Teodósio, and Morais 2021) or collect bird watches (Sullivan et al. 2014). However, they still need to be standardized and coordinated to systematically monitor temporal trends and related biodiversity dynamics that are controlled by ecological networks within rural metapopulations.

Further opportunities to indicate backyard drivers are indirect measurements from remote sensing technologies that are capable of quantifying surface properties on the basis of varying spectral information induced by interacting and recorded solar radiation. Remote sensing provides time series of satellite imagery that map spatially explicit reflectance signatures of plant species and habitats, vegetation optical traits, and surface material compositions or even of direct chemical, structural, or taxonomic diversity approximations (Laliberté, Schweiger, and Legendre 2020; Rocchini et al. 2018; Rossi et al. 2021). Image analysis and derived information on vegetation characteristics is already widely applied for nature conservation purposes to monitor habitats in protected area networks (Nagendra et al. 2013), advance biodiversity science (Cavender‐Bares et al. 2022), enhance functional ecology (Asner and Martin 2016), or assess the impacts of land use change (Haines‐Young 2009). Remote sensing of backyards is currently limited to mapping the distribution of private gardens (Baker and Smith 2019; Mathieu, Freeman, and Aryal 2007) and to classifying land cover units using high‐resolution imagery (Wagner and Egerer 2022) in urban green areas. However, there is still a lack of tracking change of complex backyard structures utilizing image time series analyses, particularly, including backyards in rural settlements of CEE countries (Shahtahmassebi et al. 2021).

### 4.2 An Example of Backyard Process Delineation Using Remote Sensing Proxies

Backyard locations can be sampled using satellite time series to identify spatially and temporally explicit trends that indicate patterns and dynamics of backyard processes, essentially to identify potential causes of biodiversity change. Gardens, for example, are distinctively defined green backyard features that can be used to contrast anthropogenically managed habitats with undisturbed vegetation of the local surrounding rural landscape. For this purpose, satellite based vegetation trends were modeled between 1990 and 2024 using the normalized differenced spectral vegetation index (NDVI) (Tucker 1979) to map plant biochemical and physiological variation. Garden areas are therein characterized by trends of greening when the NDVI is significantly increasing and by trends of diversification when the within year NDVI variance is significantly increasing. The trend significance is calculated after detrending known background trends of, e.g., greening (Piao et al. 2020) or phenology shift (Cleland et al. 2007) using abandoned land use areas as reference for unmanaged habitats. We used an area‐wide biotope type mapping for the federal state of Brandenburg to extract the polygon centroids of garden areas and closest abandoned land use polygons (Figure 2). For each centroid all available annual Landsat 5 & 8 NDVI values within the growing season between May and September only around villages and hamlet centers (radius = 1 km retaining 24.197 centroids) were extracted. In each growing season from 1990 to 2024 the average (greening) and the variation (variance) of NDVI values were derived for each centroid and modeled as temporal regression after removing local trends by subtracting greening and variance trends of closest abandoned land use polygons. Thus, a landscape decoupling of garden areas can be proven if the slope of the respective regression line is statistically significant (*p* < 0.05) after detrending. Due to differing sensor characteristics, we analyzed the two sensors separately resulting in two time periods 1990–2011 (Landsat 5) and 2013–2024 (Landsat 8). For the area of Brandenburg, it reveals that from 1990 to 2011 17.4% and from 2013 to 2024 10.93% of all garden centroids exhibit two significant trends, in both greening and diversification, while in 71.05% (1990–2011) and in 76.78% (2013–2024) of all cases at least one trend is significantly different from the local background trend, which indicates a strong tendency of garden decoupling in the considered time periods.

**FIGURE 3:**
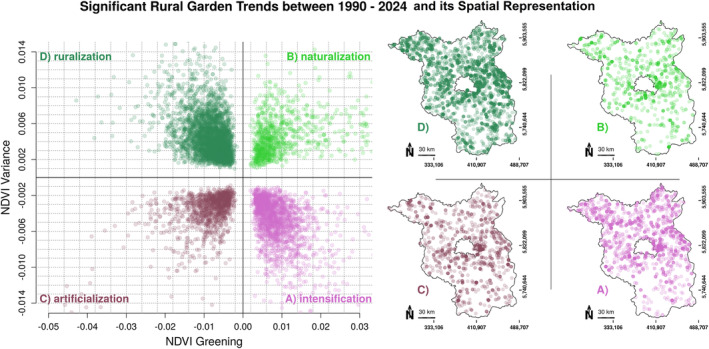
Significant (*p* < 0.05) Landsat 5 & 8 NDVI greening and within‐year variance trends from 1990 to 2024 of background detrended rural garden centroids showing *n* = 6266 gardens (25.9% of all gardens that have two significant trends in at least one time period 1990–2011 [Landsat 5] and 2013–2024 [Landsat 8]); axes show the slopes of the detrended NDVI regression lines; on the maps each point represents an actual garden location with significant trend that can be assigned to one of the 4 trend clusters (A–D) and spatially mapped to reveal spatial structures in the federal state of Brandenburg, Germany.

Significant NDVI trends for garden areas can additionally be partitioned into clusters according to the trend direction and strength that are defined by the algebraic sign and magnitude of the slope derived from the detrended NDVI regression line (Figure 3). Based on these slopes of greening and within‐year NDVI variation, garden areas can be assigned to clusters that are postulated to represent temporal backyard dynamics of (A) intensification that is characterized by decreasing NDVI variability due to plant species homogenization in, e.g., ornamental lawns or coniferous hedges and a positive greening trend due to irrigation, agrochemical usage or land‐use conversion, of (B) naturalization that also shows a positive greening trend when paved or bare grounds are getting replaced by new vegetation canopies but also indicating an increased species diversity represented by higher phenological variation, of (C) artificialization that follows a reduction of green features and is further accompanied by decreasing species and phenological variation due to, e.g., sealing into homogenous paved grounds and of (D) ruralization that entails negative greening trends due to variable garden usage for, e.g., private farming or animal keeping, which additionally increases seasonal variability of NDVI signals due to small‐scale land use patterns.

Interestingly, the relative contribution of designated hypothetical process categories (A–D) is not stable over time, e.g., artificialization was important from 1990 to 2011 (21%) and reduced from 2013 to 2024 (8%) while naturalization was doubled from 9% to 18% comparing both time periods. Further quantitative validation is however needed to derive a realistic evaluation of actual backyard management as proposed in the research agenda. Irrespective of process definitions, NDVI trend analysis reveals both, garden trends that are not in accordance with expected regional variations caused by, e.g., climate change dynamics and gardens that form clusters in which a coherent history of management activities is represented. Thus, human‐environmental interactions can be delineated as impacts that are decoupled from quasi‐natural process dynamics. NDVI clusters can additionally occur as spatial patterns in terms of local‐structural similarities (e.g., north–south intensification gradient Figure 3) or as random spatial distributions making each backyard part of a unique individual behavior. It illustrates that backyard dynamics in rural settlements can be made visible over time and in space whereby the underlying mechanisms of a changing rural metapopulation systems are highly influenced by individual living conditions that can vary significantly within the neighborhood and hence creates multiple overlapping temporal dynamics at spatially small scales. In the future, long‐term retrospective image analyses using VIs will be based on the next generation spatially highly resolved satellite time series (0.5–3 m) that will allow for a more accurate delineation of different backyard features, particularly extracting building structures and differentiating between management practices and habitat types and their spatial distribution. In this regard further indicators to monitor backyard metapopulations via remote sensing proxies need to be defined that extract information from phenological traits comparing seasonal harmonic parameters (e.g., amplitude, length, and shift) (Donnelly, Yu, and Liu 2021; Tian et al. 2020), from plant community composition discriminating species and habitats (Shahtahmassebi et al. 2021), from direct diversity estimations mapping spectral heterogeneity (Rocchini et al. 2010) or from morphological features extracting surface texture, vegetation structure and building architectural style (Goodwin et al. 2009; Ossola et al. 2019). Finally, time series analyses must include varying socioeconomic stages of development to reveal interdependencies of human behavior and biodiversity responses in a rural landscape, particularly to foster region‐based measures to maintain and recreate a connected backyard metapopulation.

## 5 Conclusion

### 5.1 Toward a New Socioecological Mindset

There are evidently versatile pressures on the diversity of species and habitats emerging from the backyards of rural settlements. Their number and spatiotemporal dynamics may outrank effects following agricultural intensification, particularly in an interconnected rural landscape of the past 30 years. Measuring and monitoring rural hotspots will provide new indicators to disentangle significant drivers and to build future scenarios that describe recent trends of biodiversity decline as response to a changing rural metapopulation in old cultural landscapes. In this regard, backyard research, exemplifies direct links between human‐environmental interactions and hence opens up new options for private engagement, participation, and active involvement in nature conservation. It deploys key feedback loops, which reveals biophysical implications of backyard management and thus enable a reflection of story lines to control biodiversity (Dougill et al. 2006). Feedback learning can motivate to adapt social norms toward sustainable and environmental‐friendly practice, advance ecological outcomes of individual behavior and finally to empower community diffusion through new socioecological standards (Cook, Hall, and Larson 2012; Goddard, Dougill, and Benton 2013). We do not always need to change the world by addressing heteronomous actions such as agricultural land use practice or large area protection networks. In fact, we can change our backyard behavior, therein stimulate environmental education and increase the awareness for local diversity to finally enable new knowledge about backyard biodiversity and its implications for future conservation strategies (Kim and Byrne 2006). There is already evidence that urban green spaces can support the conservation of mammals, avifauna or insects (Daniels and Kirkpatrick 2006; Hunter and Hunter 2008; Van Helden, Close, and Steven 2020) while creating interconnected habitats within the residential ecosystem (Goddard, Dougill, and Benton 2010). However, there is an apparent lack of rural hotspot research considering effects of changing socioeconomic conditions on village life and associated backyard processes that highly control patterns of connectivity and integrity in the broad context of old cultural landscapes.

## Author Contributions

**Carsten Neumann:** conceptualization (lead), data curation (lead), formal analysis (lead), funding acquisition (equal), investigation (lead), methodology (lead), visualization (lead), writing – original draft (lead), writing – review and editing (lead). **Robert Behling:** conceptualization (equal), data curation (supporting), formal analysis (equal), writing – original draft (equal), writing – review and editing (equal). **Gabriele Weiss:** conceptualization (supporting), supervision (supporting), validation (equal), writing – original draft (supporting), writing – review and editing (supporting).

## Conflicts of Interest

The authors declare no conflicts of interest.

## Data Availability

The data that support the findings of this study, i.e., Landsat 5 & 8 NDVI time series from 1990 to 2024 for gardens, shapefiles of garden locations & villages centers, metadata, and R‐scripts to freely access, process, and analyze the entire workflow as described in Section 4 (Figure 3) are provided here: https://doidata.gfz.de/Rural_Hotspots_C_Neumann_2024/NDVI_for_Gardens.zip.
